# Recent Insights into Roles of Hypoxia-Inducible Factors in Retinal Diseases

**DOI:** 10.3390/ijms251810140

**Published:** 2024-09-21

**Authors:** Deokho Lee, Yohei Tomita, Yukihiro Miwa, Hiromitsu Kunimi, Ayaka Nakai, Chiho Shoda, Kazuno Negishi, Toshihide Kurihara

**Affiliations:** 1 Laboratory of Photobiology, Keio University School of Medicine, Tokyo 160-8582, Japan; 2 Laboratory of Chorioretinal Biology, Keio University School of Medicine, Tokyo 160-8582, Japan; 3 Department of Ophthalmology, Keio University School of Medicine, Tokyo 160-8582, Japan; 4 Aichi Animal Eye Clinic, Aichi 464-0027, Japan; 5 Department of Ophthalmology, Nihon University School of Medicine, Tokyo 173-8610, Japan

**Keywords:** retinal ischemia, hypoxia, ischemia

## Abstract

Hypoxia-inducible factors (HIFs) are transcriptional factors that function as strong regulators of oxygen homeostasis and cellular metabolisms. The maintenance of cellular oxygen levels is critical as either insufficient or excessive oxygen affects development and physiologic and pathologic conditions. In the eye, retinas have a high metabolic demand for oxygen. Retinal ischemia can cause visual impairment in various sight-threating disorders including age-related macular degeneration, diabetic retinopathy, and some types of glaucoma. Therefore, understanding the potential roles of HIFs in the retina is highly important for managing disease development and progression. This review focuses on the physiologic and pathologic roles of HIFs as regulators of oxygen homeostasis and cellular metabolism in the retina, drawing on recent evidence. Our summary will promote comprehensive approaches to targeting HIFs for therapeutic purposes in retinal diseases.

## 1. Oxygen and Hypoxia-Inducible Factors

Hypoxia-inducible factors (HIFs) are known as strong transcription factors for oxygen homeostasis. In 1992, Gregg Semenza and Guang Wang found that a nuclear factor induced by hypoxia via de novo protein synthesis could bind to the human erythropoietin (EPO) gene enhancer at a site for transcriptional activation using Hep3B cells [1]. After three years (in 1995), Guang Wang and Gregg Semenza conducted purification and characterization of HIF-1 (120-kDa HIF-1α and 91–94-kDa HIF-1β) from EPO-producing Hep3B cells and non-EPO-producing HeLa S3 cells under hypoxic and pseudohypoxic (cobalt chloride; CoCl_2_) conditions [2]. Around the same time (in 1991), similar findings were summarized by a research group from Peter Ratcliffe using the mouse *Epo* gene [3]. Meanwhile, there was a research group (including William Kaelin) studying von Hippel–Lindau (VHL) syndrome [4]. They suggested negative regulation of hypoxia-inducible genes (e.g., vascular endothelial growth factor, VEGF) by the VHL protein [5,6]. A series of pioneering research findings from Gregg Semenza, Peter Ratcliffe, and William Kaelin have been acknowledged as the discovery of how cells and tissues sense oxygen.

## 2. The Signaling of Hypoxia-Inducible Factors

**Figure 1:**
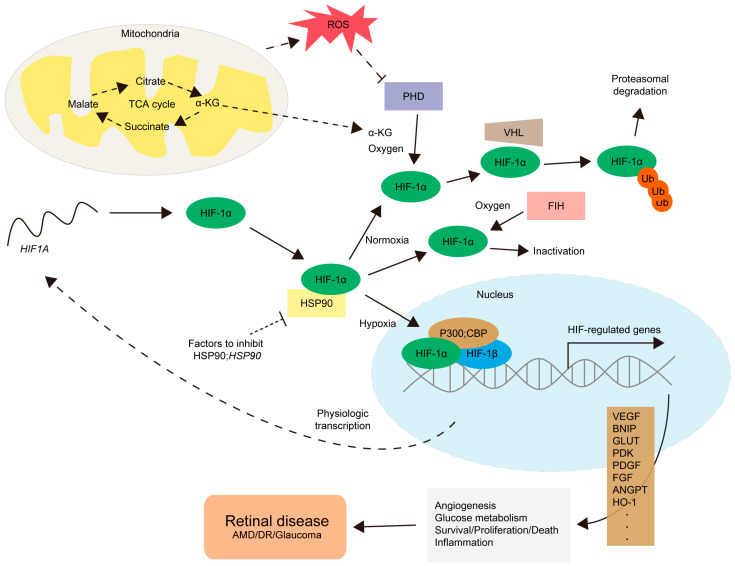
The regulation of HIF-1α and its target gene expressions. For the physiological process, the mRNA for the HIF1A gene is converted into the HIF-1α protein by ribosomes (a long curved dotted line, starting from the nucleus to the *HIF1A* mRNA). Under a normoxic condition, prolyl hydroxylases (PHDs), relying on α-ketoglutarate (α-KG) and oxygen levels, hydroxylate HIF-1α, leading to von Hippel–Lindau (VHL)-mediated proteasomal degradation with ubiquitination (Ub). Factor-inhibiting HIF-1 (FIH) also hydroxylates HIF-1α to prevent necessary interactions with its co-factors including p300 or CREB-binding protein (CBP), leading to HIF-1α inactivation. Under hypoxic and/or various stress conditions, the activities of PHD and FIH are inhibited. Heat shock protein 90 (HSP90) also contributes to the regulation of the accumulation of HIF-1α under specific conditions. Un-hydroxylated HIF-1α could translocate to the nucleus, form a complex with HIF-1β and p300;CBP, and activate the transcription of HIF-regulated genes. HIF-related genes (such as vascular endothelial growth factor VEGF, platelet-derived growth factor PDGF, fibroblast growth factor FGF, pyruvate dehydrogenase kinase PDK, BCL2/adenovirus E1B 19 kDa protein-interacting protein BNIP, glucose transporter GLUT, pyruvate dehydrogenase kinase PDK, angiopoietin ANGPT, or heme oxygenase-1 HO-1) are involved in angiogenesis, glucose metabolism, survival/proliferation/death pathways, and inflammation. This finally promotes the development and progression of retinal diseases including age-related macular degeneration (AMD), diabetic retinopathy (DR), and glaucoma. Stress conditions include abnormal increases in mitochondrial reactive oxygen species (ROS) levels or metabolic dysregulations in the citric acid cycle (the TCA cycle), one of the essential energy-producing pathways occurring in mitochondria. More studies on the mode of action of HIF activation or inhibition are highly needed depending on the cell types in the retina, choroid, and retinal pigment epithelium (RPE).

HIFs are composed of α and β subunits. To date, three isoforms of HIFs have been found: HIF-1, HIF-2, and HIF-3. Their domain structures (including oxygen-dependent degradation, N-terminal transactivation, or C-terminal transactivation) were reviewed and discussed in our previous review article [7]. Under normoxic conditions, HIF-α is rapidly recognized and degraded by HIF-prolyl hydroxylases (PHDs), VHL, an E3 ligase, and a ubiquitin-mediated proteasome. Other mechanisms for HIF-α regulation include an interaction with the p53 tumor suppressor gene [8] or the molecular chaperone HSP90 gene [9]. With a very short half-life, detecting the HIF-α protein is relatively difficult under normoxic conditions. On the contrary, decreasing concentrations of oxygen (the hypoxic condition) lead to a decreased rate of HIF-α degradation because of the suppression of prolyl hydroxylation. This results in the HIF-α protein being detectable in hypoxic cells or tissues. Increased levels of the HIF-α protein lead to it being translocated into the nucleus and forming a heterodimer with the β subunit to activate various hypoxia-responsive gene expressions. The expressed genes, including VEGF, platelet-derived growth factor (PDGF), fibroblast growth factor (FGF), pyruvate dehydrogenase kinase (PDK), BCL2/adenovirus E1B 19 kDa protein-interacting protein (BNIP), or glucose transporter (GLUT), are involved in angiogenesis, inflammation, glucose metabolic adaptation, or cell death/survival/proliferation [10,11,12,13,14]. The outcomes of this process have been known to promote the development or progression of various metabolic diseases including ischemic retinopathy (Figure 1). This will be described in more detail in the following sections.

## 3. Hypoxia and Retinal Development

Retinal development is highly affected by hypoxia. As murine retinas do not have complete retinal vasculature right after birth, researchers have used murine retinas to study the roles of hypoxia in vasculature development [15,16,17]. The notion (that vasculature development follows neuronal maturation in the retina) is well described in previous reviews [18,19]. Briefly, from P1 to P10, superficial vascular plexus forms, while deep vascular plexus is completed from P8 to P12 [20]. Then, intermediate vascular plexus forms last from P14 to P20. At the developmental stage, the *Vegf* gene is found to be detected in avascular areas in the retina [19]. HIF-1α is abundantly detected in cells of the neuro-retina located in the inner nuclear layer, and vascular development in the developing retina is impaired by neuro-retina-specific (*Pax6*) knockout of HIF-1α [21]. This phenotype is mainly related to decreased endothelial tip cell numbers and reduced vessel branching. Taken together, the HIF/VEGF axis is important for retinal development.

## 4. Roles of Hypoxia-Inducible Factors in Age-Related Macular Degeneration

Age-related macular degeneration (AMD) is a progressive retinal disorder damaging the macula. Generally, two types of AMD are listed as follows: dry (atrophic AMD) and wet (neovascular AMD). Various risk factors are well discussed in a previous review [22]. Briefly, natural aging, lipid metabolism, and hypoxic responses are mainly suggested. Findings from human and murine samples have shown that HIFs might be highly involved in the development or progression of AMD.

### 4.1. Choroidal Neovascularization

A murine model of laser-induced choroidal neovascularization (CNV) has been enormously used to study the pathologic mechanisms of neovascular AMD [23]. Several laser injuries cause the perforation of Bruch’s membrane, finally resulting in subretinal blood vessel recruitment from the choroid. The time point of a maximized CNV volume has been generally suggested as day 7 after laser induction. We also found reliable CNV formation 7 days after laser induction in adult mice [24,25]. Furthermore, increases in HIF-1α protein levels were detected in the retina and the choroid/retinal pigment epithelium (RPE) 3 days after laser induction. This implies that HIF regulation in the retina or the choroid/RPE might affect CNV formation. Reductions in CNV volumes were detected in RPE-specific or neural retina-specific *Hif1a* conditional knockout mice [24]. Similar effects were found by another research group from South Korea [26]. Intravitreal injection of LbCpf1 (a CRISPR RNA-guided endonuclease) targeted to *Vegfa* or *Hif1a* using adeno-associated virus (AAV) showed efficient gene disruption rates in ocular cells, and LbCpf1 targeted to *Vegfa* or *Hif1a* in RPE cells extensively reduced CNV volumes. Lin et al. also demonstrated the therapeutic impacts of HIF-1 knockout in the RPE on laser-induced CNV, with reductions in HIF-1α and VEGF protein levels in the eyecup [27].

Recently, a murine model of subretinal lipid peroxide-induced CNV has been used to study the roles of HIF-1 in neovascular AMD [28]. Lipid peroxide causes oxidative stress in the RPE, resulting in increases in HIF-1α expression 3 days after the injection. CNV formation is observed 14 days after the injection, with increases in *Vegf* and *Angptl4* mRNA expressions. Under this condition, an HIF-1 inhibitor digoxin treatment reduced CNV formation. They reported that increases in oxidative stress (analyzed with the protein expression of thioredoxin) are detected in human AMD eyes (especially, the RPE). Oxidative stress (such as 4HNE: 4-hydroxynonenal, tBH: tert-Butyl hydroperoxide, and H_2_O_2_: hydrogen peroxide) increases HIF-1α expression in RPE cells. Using human-induced pluripotent stem cell (hiPSC)-derived RPE cells, the notion that oxidative stress promotes the accumulation of HIF-1α and HIF-1α-regulated gene expression was further confirmed. Ji Cho et al. demonstrated that oxidative stress-mediated thioredoxin-interacting protein (TXNIP) loss causes RPE dysfunction [29]. The knockdown of TXNIP increases HIF-1α expression, which further alters the enhanced secretion of VEGF (from mRNA expressions to protein levels) from RPE cells and the stimulation of angiogenesis in co-cultured human retinal microvascular endothelial cells (HRMECs).

### 4.2. Subretinal Fibrosis

Subretinal fibrosis is one of the pathologic outcomes of a wound healing response against CNV in neovascular AMD. Several molecular mechanisms and clinical implications of subretinal fibrosis are well described in previous reviews [30,31]. Recently, we further found that subretinal fibrosis formation is highly related to regulations of HIFs in the RPE [32]. Conditional knockout of *Hif1a* and/or *Hif2a* in the RPE reduces laser-induced subretinal fibrosis formation 5 weeks after laser damage. Expected opposite effects were seen in RPE-specific *Vhl*-conditional knockout mice. In human AMD eyes, HIF-1α expression is strongly detected, while the expression of HIF-2α is not. Along with this, the notion that HIF-1α regulates p53 stability and activates miRNA-34a transcription to promote fibrosis formation was applied to the aspect of subretinal fibrosis. Xie et al. showed that HIF-1α promotes p53 stability and its nuclear translocation in RPE cells [33]. HIF-1α-mediated p53 activation increases miRNA-34a expression. The HIF-1α/p53/miRNA-34a axis facilitates hypoxia-induced RPEs’ epithelial–mesenchymal transition (EMT) process. Blocking this axis modulates laser-induced CNV and subretinal fibrosis formation. Taken together, HIF-1α might be the main contributor for the development of subretinal fibrosis.

### 4.3. Photoreceptor Loss

Photoreceptor loss is one of the pathologic features of AMD eyes [34]. Aging, choriocapillaris flow reduction, or light damage might provide reasons for photoreceptor loss [35,36,37]. Barben et al. demonstrated that hypoxia-responsive genes were highly expressed in the aged human retina [38]. HIF-1-dependent rod photoreceptor degeneration was found using the inactivation of the *Vhl* gene in mouse rods with additional inactivation of the *Hif1a* and/or *Hif2a* gene. Clear detection of photoreceptor damage needs around 6 months of aging, which represents slowly progressing chronic retinal degeneration. RPE damage was also detected in rod-specific inactivation of *Vhl* mice. The treatment of AAV-mediated anti-*Hif1a* shRNA could protect against retinal degeneration. Samardzija et al. examined retinal physiologic changes by cone-specific *Hif1a* or *Hif1a* and *Hif2a* knockout and found that retinal morphology or function was not changed by the knockout [39]. Furthermore, using a model of light-induced retinal degeneration, they concluded that cones may not essentially need HIFs to survive under the pathological condition. Taken together, the modulation of HIFs in rods might be more important than that in cones for retinal protection.

### 4.4. Metabolic Dysregulation

Then, how does chronic activation of HIFs in rods affect retinal metabolism? Todorova et al. found that chronic HIF activation in rods affects lactate production, the oxidative phosphorylation (OXPHOS) pathway, compositions of mitochondrial proteins, and the citric acid cycle [40]. A series of metabolic dysregulations could cause shortening of the outer segments in photoreceptors. This work gives important insight into photoreceptor protection under HIF-mediated pathologic conditions.

Retinal energy metabolism is highly influenced by vessel supply in physiology and pathology. This aspect is well discussed in previous reviews from the Lois Smith group [18,41]. In particular, Joyal et al. demonstrated that reduced levels of pyruvate, acetylcarnitine, and α-ketoglutarate are seen in the fuel-deficient *Vldlr* knockout retina [42]. As PHDs are α-ketoglutarate-dependent together with oxygen (Figure 1), the degradation process of HIFs could be disrupted. This leads to increases in VEGF levels. Heckel et al. further proposed that excess dietary lipids could restrain autophagy, negatively affecting energy metabolism. This outcome might induce dysregulations in HIF expression, finally leading to pathologic neovascularization [43]. Taken together, energy metabolism should be further considered to regulate pathologic HIFs in the eye.

### 4.5. Others

Recently, we found that extensive light exposure upregulated HIF-1α expression in the retina [44]. Retinal HIF-1α and HIF target gene BNIP3 expressions increased 24 h after light exposure. After 7 days of light exposure, retinal dysfunction and histologic damage including apoptosis in the outer retina were detected. Under this condition, the systemic treatment with halofuginone (a novel HIF inhibitor) could reduce light-induced retinal damage. This implies that HIF inhibition might be beneficial for the prevention of light-induced retinopathy. However, we could not rule out the other reported systemic effects of halofuginone on inflammation in our system. This needs more investigations. Meanwhile, hypoxic preconditioning has been described to protect photoreceptors against light-induced damage [45,46]. The other study suggested that HIF-1 might promote retinal photoreceptor survival in a mouse model for ocular oxidative stress induced by the treatment of sodium iodate (NaIO_3_) [28]. To understand the clear roles of HIF activators or inhibitors on outer retinal damage, more studies in terms of the drug dosage and administration time points and methods should be required depending on the experimental model.

## 5. Roles of Hypoxia-Inducible Factors in Diabetic Retinopathy

Diabetic retinopathy (DR) is one of the most frequent complications of diabetes mellitus. Proliferative diabetic retinopathy (PDR), characterized by the presence of abnormal new vessels that grow outside the retina, is considered the most serious form of DR [47]. Various risk factors for DR development are well discussed in our previous review [48]. Briefly, hyperglycemia-induced oxidative stress, inflammation, advanced glycation end products, hypoxic insults, and the protein kinase C pathway have been found to be associated with reactive gliosis, vascular damage and leakage, and neuronal retinal cell death. The impacts of systemic regulations of HIFs and the related regulatory proteins in diabetic macular edema and DR have been gradually reported in clinics [49,50]. As diabetes affects the whole body’s physiological systems and hormones, the regulation of HIFs in one tissue for treatment could be detrimental on other tissues. Therefore, in-depth studies on HIF regulation in DR must be carefully performed.

Two main phenotypes are generally considered in experimental DR research: inflammatory vascular damage and retinal neuronal damage. To study neovascularization, murine models of oxygen-induced retinopathy (OIR) have been widely used [51,52], although OIR mice are not a direct model for DR. For diabetes-induced retinal damage, streptozotocin (STZ) is widely used in preclinical studies [53].

### 5.1. Oxygen-Induced Retinopathy

For OIR research, neonatal mice and a nursing mother are kept in room air from birth through P7 [54]. At P7, pups (with the mother) are exposed to 75% oxygen. Hyperoxia inhibits retinal vessel growth and causes vessel loss. Mice are returned to room air at P12. This induces neovascularization and vessel regrowth in the retina. Neovascularization is maximally formed at P17. The relative hypoxia occurs due to the imbalance between neuro-retinal and retinal vascular development. Depending on the laboratory condition, experimental conditions (oxygen levels or pups’ age) need minor adjustments [55]. In our previous study, the mRNA expressions of *Hif1a* and HIF-related genes (*Vegf*, *Pdk1*, and *Bnip3*) were upregulated at P17 [56]. An increase in HIF-1α protein expression was also detected at P17. Under this condition, topotecan (a HIF inhibitor) treatment suppressed neovascularization, with a reduction in HIF-1α protein expression.

Zhang et al. showed different time points of the upregulation of HIFs [57]. HIF-1α expression maximally increases by P13, while its expression is not clearly detected in the inner retina at P14. The expression of HIF-2α is detected in the inner retina at P14 and persists with a slight decrease until P17. This implies that HIF-1α might have a rapid but transient accumulating feature and HIF-2α might have the opposite. Furthermore, they showed that pharmacologic and genetic inhibition of HIFs could reduce neovascularization in OIR mice.

Xin et al. focused on the roles of Müller glia in OIR mice [58]. They found that hypoxic Müller glia promotes vascular permeability in OIR mice by stabilizing HIF-1α expression. Furthermore, Müller glia’s *Angptl4* is identified as one of the key cytokines upregulated from the HIF signaling pathway. Those findings are further supported by retinal tissues from subjects with diabetic eye disease; the localization of HIF-1α and ANGPTL4 to ischemic Müller glia.

Huang et al. focused on the roles of mitochondrial dynamics in retinal neovascularization [59]. VEGF treatment induces mitochondrial fission in endothelial cells through the phosphorylation of dynamin-related protein 1 (DRP1). DRP1-dependent mitochondrial fission is found to be involved in angiogenesis in endothelial cells. Mitochondrial fission is further important for producing ROS. Mitochondrial ROS modulates HIF-1α-dependent glycolysis and angiogenic switch in endothelial cells. The inhibition of DRP1 improves retinal neovascularization in OIR mice, with a reduction in HIF-1α expression.

With the notion that HSP90 regulates the VHL-independent HIF-1α-degradative pathway [60], Jo et al. examined whether HSP90 inhibition-mediated HIF-1α destabilization could affect retinal neovascularization [61]. Using novel HSP90 inhibitors (SH-1242 and SH-1280), they found the suppression of neovascularization in OIR mice. Furthermore, these HSP90 inhibitors do not show dramatic toxicities toward retinal tissues, which could be used as alternatives to anti-VEGF agents. Taken together, along with the evidence from human samples, the pathologic roles of HIFs in retinal neovascularization have been extensively studied using OIR models.

### 5.2. Streptozotocin-Induced Diabetes

Although OIR models are widely used to study the mechanisms of neovascularization in the retina, they do not exhibit hyperglycemia. To clarify the complicated pathologic effects of hyperglycemia in retinal neuronal damage and vascular dysfunction in DR, chemically induced STZ diabetic models are popularly used in basic mechanism studies and therapeutic drug screening. Although a large number of experimental DR studies have been performed using STZ mice or rats, their pathologic ocular changes (e.g., inner retinal and/or outer retinal damage) and the observation time points (e.g., several weeks and/or months after the STZ injection) are still under debate.

Several STZ injection protocols are suggested to induce experimental diabetes in mice. A single high dose (200 mg/kg) or repeated low doses of STZ (40 mg/kg for 5 days) could be used [62]. The single high dose of STZ might increase death rates of STZ mice as it is a toxic agent, while the low doses of STZ could have higher failure rates of STZ mouse induction. To make successful STZ mice without failure or unexpected death, this value is also adjusted according to each laboratory condition [63].

Recently, Zhang et al. clearly characterized the upregulation of HIFs and HIF-regulated angiogenic factors with vascular permeability in STZ-induced diabetic mice [64]. Increased vascular permeability was detected in STZ mice for 6 months. Although hypoxia was not detected in the retina up to 9 months, increases in HIF-1α and HIF-2α protein levels were clearly observed in the retina of STZ mice for 6 months, analyzed by Western blotting. Retinal HIF-1α, VEGF, or ANGPTL4 expression starts to be detectible from approximately 1 to 3 months of hyperglycemia, analyzed by immunohistochemistry. Furthermore, treatment with HIF inhibitors could reduce vascular permeability.

Liu et al. also aimed to determine the increase in HIF-1α expression in STZ mice [65]. Based on their Western blotting data, 8 weeks are needed to clearly detect HIF-1α expression. Furthermore, under this condition, turbulent arrangements of the retinal nerve fiber layer and retinal ganglion cell layer are slightly detected. Lin et al. further found that genetic HIF-1α disruption in Müller cells modulates retinal vascular leakage and adherent leucocytes under the STZ-induced diabetic condition [66]. Taken together, chronic hyperglycemia under the experimental STZ condition also clearly increases the expression of pathologic HIFs and further damages retinal vessels.

As long-term mouse experiments are needed for retinal HIF studies under the STZ-induced diabetic condition, external metabolic, surgical, or genetic combination cues might be further considered to improve or promote DR research.

## 6. Roles of Hypoxia-Inducible Factors in Glaucoma

Glaucoma is a heterogenous condition of progressive optic neuropathies characterized by retinal ganglion cell (RGC) loss or RGCs’ axonal degeneration [67]. Increased intraocular pressure (IOP) is one of the major risk factors for the development of glaucoma, while normal-tension glaucoma also exists [68,69]. HIF-1 activation or hypoxia might be associated with the pathologic mechanisms of glaucoma, based on the evidence of HIF upregulation in the human glaucomatous retina and optic nerve head [70].

### 6.1. Magnetic Microbead-Induced Chronic Intraocular Pressure

For the chronic IOP elevation model, magnetic microbeads were injected into the anterior chamber of mouse eyes using a glass-pulled micropipette connected to a micro-syringe pump [71,72]. Based on the data from Assraa Jassim and Denise Inman, ocular hypertension was stably maintained for more than 4 weeks, as the beads blocked the aqueous humor outflow pathway through the trabecular meshwork [73]. Gradually, RGC damage and impaired anterograde transport were detected in this model. Four weeks after the surgery, increases in *Hif1a* and *Hif2a* expressions in the retina were detected. Significant increases in HIF-1α and HIF-2α expressions were also co-localized with GFAP or IBA1 expression in the retina.

### 6.2. Retinal Ischemia/Reperfusion Injury-Induced Acute Intraocular Pressure

In terms of RGC loss induced by acute and transient IOP elevation, a retinal ischemia/reperfusion (I/R) injury mouse model is widely used [74]. IOP elevation in retinal I/R injury mice is induced by ocular anterior chamber cannulation with a thin needle flowing the drip of an elevated saline reservoir. Induction of ischemic time varies from 30 min to several hours depending on the laboratory condition. Reperfusion starts when the cannula is removed.

We found that HIF-1α protein levels were upregulated in the retina from 6 h after the injury [75]. Its expression was well detected until 24 h after the injury [76]. Inner retinal damage (regarding RGC loss and visual dysfunction) was observed approximately 1 week after the injury. Ji et al. found maximally increased levels of HIF-1α and VEGF 3 days after the injury and a gradual reduction in retinal function (analyzed by electroretinography) from day 0 to day 7 after the injury [77]. HIF-1α might have significant effects on the inner retina under the retinal I/R injury-induced pathologic condition.

Using a laser capture microdissection technique, we collected inner retinal samples from the entire retina after retinal I/R injury and found an increase in *Bnip3* (one of the HIF target genes) expression in the inner retina [78]. Along with the finding that retinal HIF inhibition has neuroprotective effects against retinal I/R injury, genetic BNIP3 inhibition shows neuroprotection (analyzed by hematoxylin and eosin staining and visual evoked potential measurement) under the same condition. This implies that the HIF/BNIP3 axis might be the pathologic pathway in retinal I/R injury. However, specific cell types with this pathologic axis together have not been clearly defined. Although HIF and its downstream consequences might play important roles in glaucoma pathology, more stacking evidence is desired, compared to the other diseases above.

## 7. Conclusions

**Figure 2:**
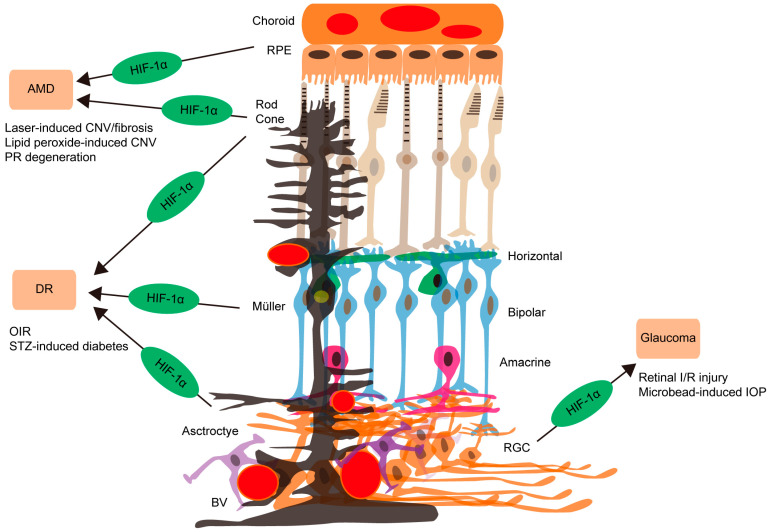
The accumulating evidence of HIF-1 involvement causing retinal diseases summarized in the current review article. The development of dry or wet age-related macular degeneration (AMD) is highly related to HIF-1α activation in retinal pigment epithelial (RPE, apricot; upper) or photoreceptor (PR, rod and cone) cells, using laser-induced choroidal neovascularization (CNV)/fibrosis, lipid peroxide-induced CNV, and PR degeneration models. On the other hand, the therapeutic roles of HIF activation depending on the disease condition should be further confirmed. For diabetic retinopathy (DR) development and progression, HIF-1α activation in photoreceptors (two types of light brown), Müller glia (black), or astrocytes (purple) is found to be important, using oxygen-induced retinopathy (OIR) and streptozotocin (STZ)-induced diabetes models. Relationships of glaucoma and retinal ganglion cell (RGC, orange; low) loss with retinal HIF-1α activation are suggested, using retinal ischemia/reperfusion (I/R) injury through a transient elevation of intraocular pressure (IOP) and microbead-induced chronic IOP models. BV (red circles): blood vessel. Horizontal cells (green). Bipolar cells (sky blue). Amacrine cells (pink).

In this review article, we summarized the important regulatory effects of HIF-1 in various retinal diseases (AMD, DR, and glaucoma), drawing on recent evidence (Figure 2). HIFs could be intriguing therapeutic molecular targets as their expressions are only highly maintained under pathologic conditions [79,80,81]. Although targeting VEGF has proven effective to treat various retinal ischemic diseases, failure cases and safety issues regarding repeated injections have made researchers shift their attention to finding an alternative and additional HIF-inhibiting strategy. HIF is the first transcription factor responsive to hypoxia and oxidative stress. It is also an upstream regulator of VEGF and other pathologic factors. This implies that inhibiting HIF could be effective for preventing pathologic phenotypes of various retinal diseases (AMD, DR, and glaucoma). However, depending on the disease condition, HIF activation is required for its physiologic and therapeutic effects. This aspect should be further investigated. Moreover, cell type-specific modulation of HIFs could be considered in that each retinal disease has distinct HIF-producing cell types in the retina (such as RPE cells, photoreceptors, glia, and RGCs). Our current summary will help form a comprehensive understanding of the regulatory roles of HIFs and promote novel therapeutic approaches for targeting HIFs to cure various retinal diseases.
